# Multiarticular chronic tophaceous gout with severe and multiple ulcerations: a case report

**DOI:** 10.1186/1752-1947-5-397

**Published:** 2011-08-19

**Authors:** Evangelos Falidas, Efstathios Rallis, Vasiliki-Kalliopi Bournia, Stavros Mathioulakis, Emmanouil Pavlakis, Constantinos Villias

**Affiliations:** 1 First Department of Surgery, 417 NIMTS Veterans Administration Hospital of Athens, Monis Petraki 10-12, Athens, 11521,Greece; 2 Department of Dermatology, 417 NIMTS Veterans Administration Hospital of Athens, Greece; 3 Department of Rheumatology, 417 NIMTS Veterans Administration Hospital of Athens, Greece

## Abstract

**Introduction:**

Gout is a common inflammatory arthritis caused by articular precipitation of monosodium urate crystals. It usually affects the first metatarsophalangeal joint of the foot and less commonly other joints, such as wrists, elbows, knees and ankles.

**Case presentation:**

We report the case of a 75-year-old Caucasian man with tophaceous multiarticular gout, soft-tissue involvement and ulcerated tophi on the first metatarsophalangeal joint of the left foot, on the first interphalangeal joint of the right foot and on the left thumb.

**Conclusion:**

Ulcers due to tophaceous gout are currently uncommon considering the positive effect of pharmaceutical treatment in controlling hyperuricemia. Surgical treatment is seldom required for gout and is usually reserved for cases of recurrent attacks with deformities, severe pain, infection and joint destruction.

## Introduction

Gout is a common disorder of uric acid metabolism, characterized by recurrent episodes of inflammatory arthritis, tophaceous soft tissue deposits of monosodium urate crystals, uric acid renal calculi and chronic nephropathy. We report the case of a 75-year-old Caucasian man suffering tophaceous multiarticular gout and soft-tissue involvement, presenting with ulcerated tophi overlying the first metatarsophalangeal joint of the left foot, the first interphalangeal joint of the right foot and the left thumb. We also emphasize the disabling effects of the under-treated hyperuremic arthropathy.

## Case presentation

A 75-year old Caucasian man with a long-standing history of tophaceous gout and several recurrent episodes of arthritis during the past five years presented with a large, painful, ulcerated tophus located on the first metatarsophalangeal joint of his left foot to our emergency department. He had intentionally interrupted treatment with allopurinol four months previously; however, he did not report any recent deviations from his standard diet, any alcohol abuse or diuretic treatment. Five days before presenting to the emergency department, a tophus on the first toe of his left foot had become painful, red and swollen. He tried a course of non-steroidal anti-inflammatory drugs (NSAIDs) without improvement. Ten hours before seeking medical assistance, the tophus burst releasing a viscous, chalk-like material.

**Figure 1:**
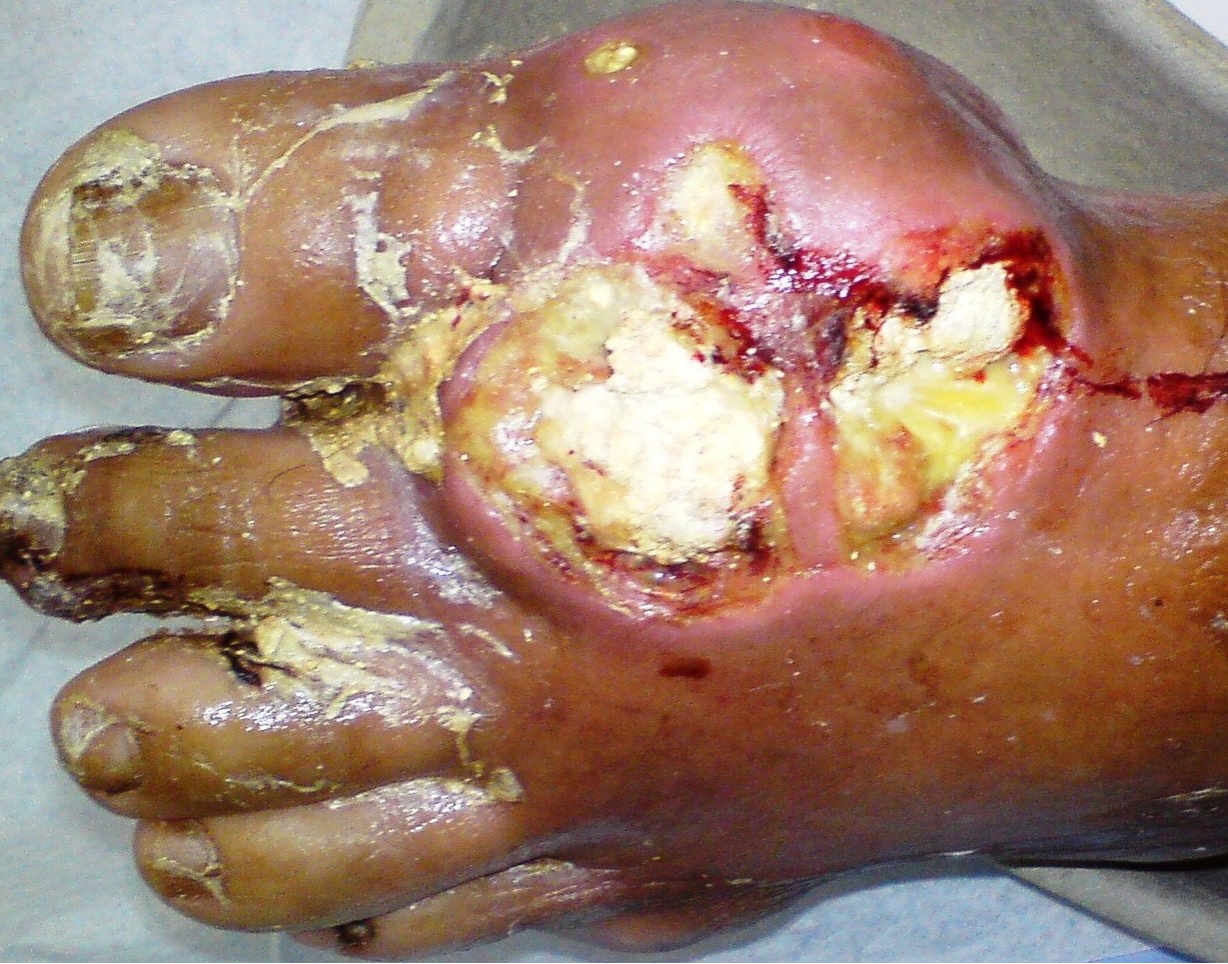
**Voluminous, erupted and ulcerated nodule on the first metatarsophalangeal joint of the left foot containing chalky material (on admission)**.

**Figure 2:**
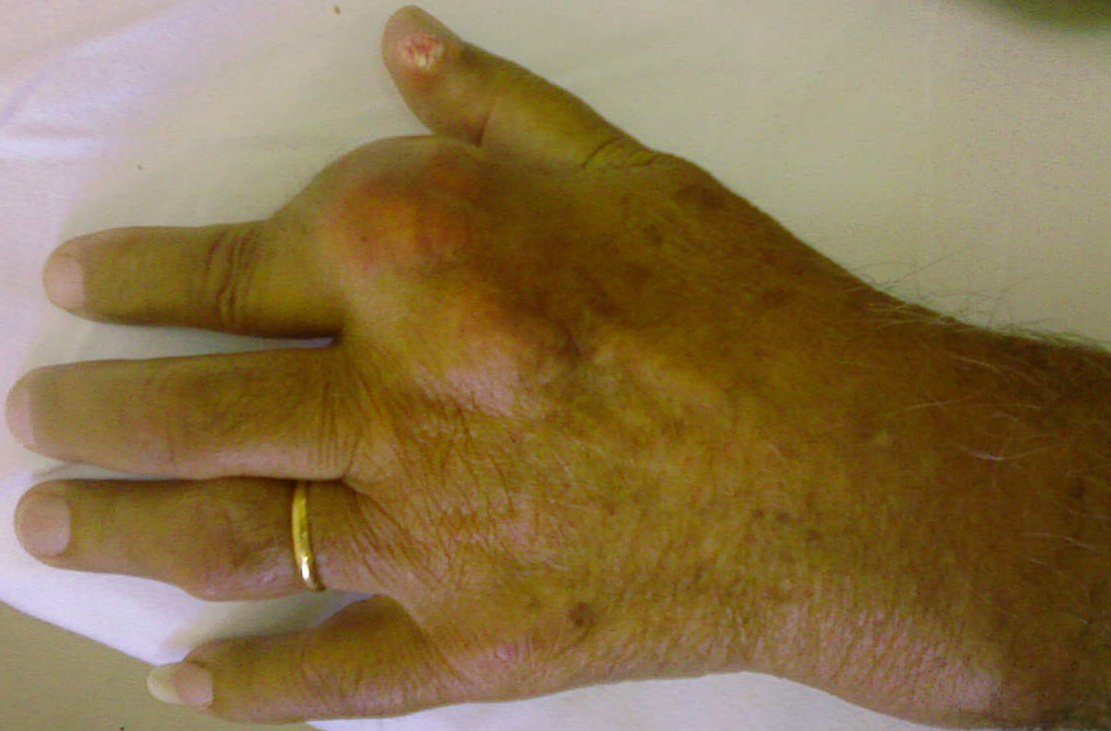
**Voluminous tophi of the first and second metacarpophalangeal joint of the left hand**. A small ulcerated tophus is also visible on the fingertip of the thumb.

**Figure 3:**
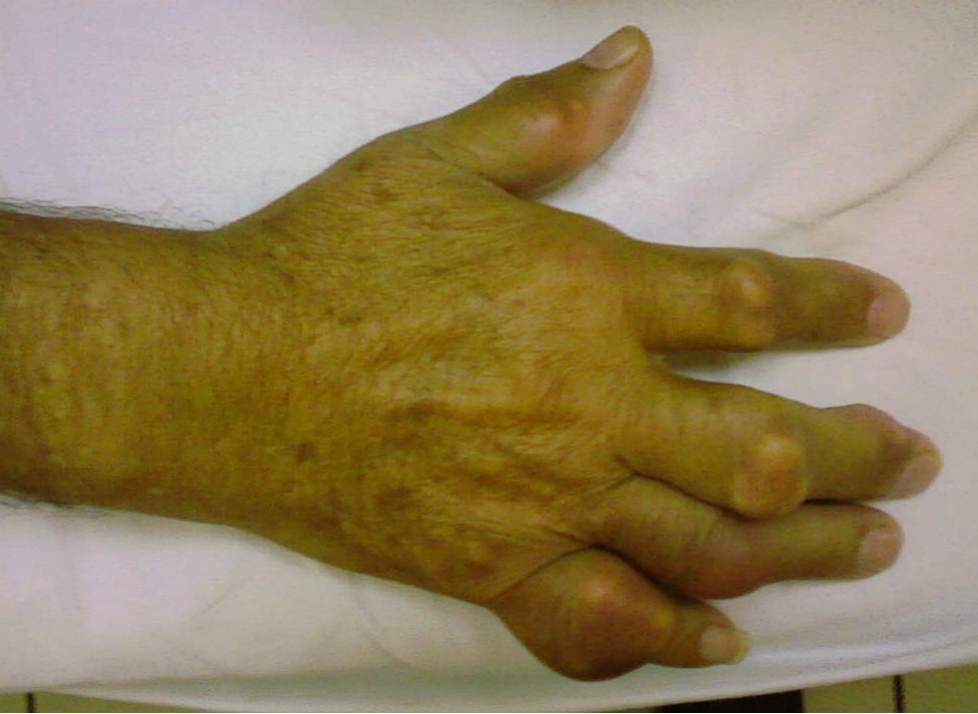
**Tophi of the interphalangeal joints of the right hand**.

**Figure 4:**
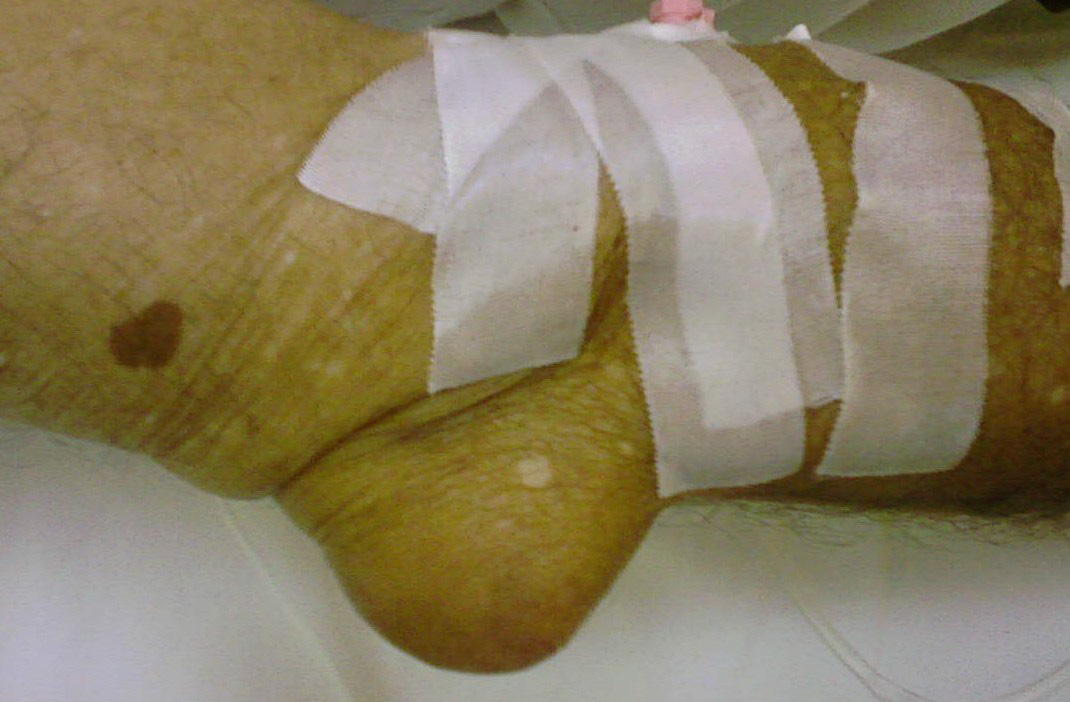
**Sizable tophus of the right elbow**.

**Figure 5:**
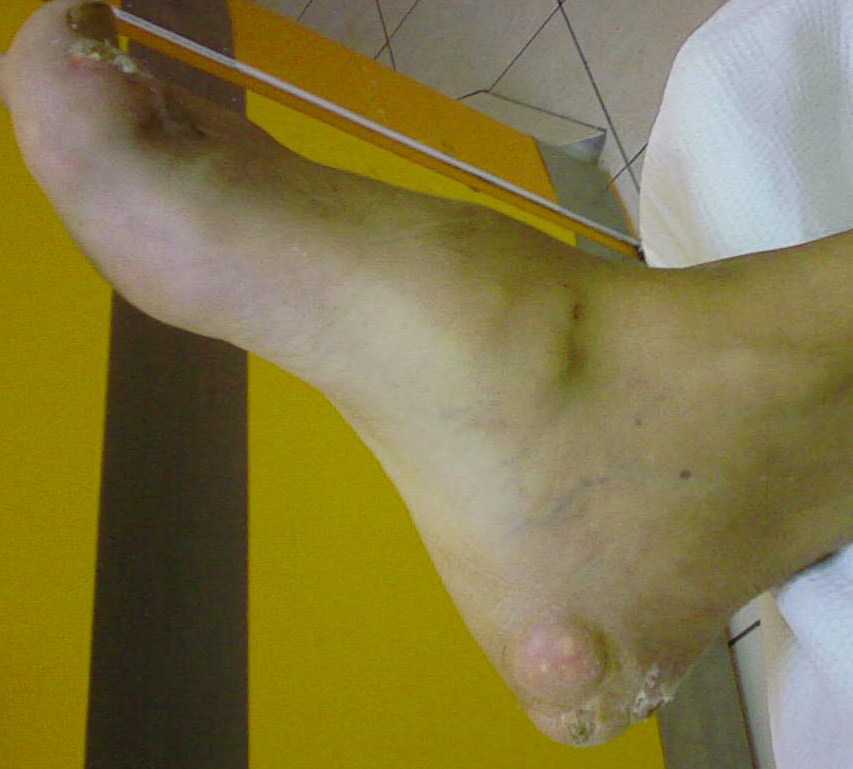
**Tophus of the medial surface of the right heel and small ulcer of the first interphalangeal joint of the right foot**.

On physical examination he had a mild fever of 37.8°C. A greyish, voluminous and ulcerated nodule containing chalky material was located on the first metatarsophalangeal joint of his left foot (Figure 1). Further examination revealed multiple other tophi overlying the first and second metacarpophalangeal joints of his left hand (Figure 2) and the interphalangeal joints of his right hand (Figure 3), wrists, elbows (Figure 4), ankles, interphalangeal and metatarsophalangeal joints of the feet and heels (Figure 5). Two smaller ulcerated tophi were also seen on the fingertip of the left thumb and over the first interphalangeal joint of the right foot. Many joints were also deformed. The first metatarsophalangeal joint of his left foot was totally nonfunctional.

**Figure 6:**
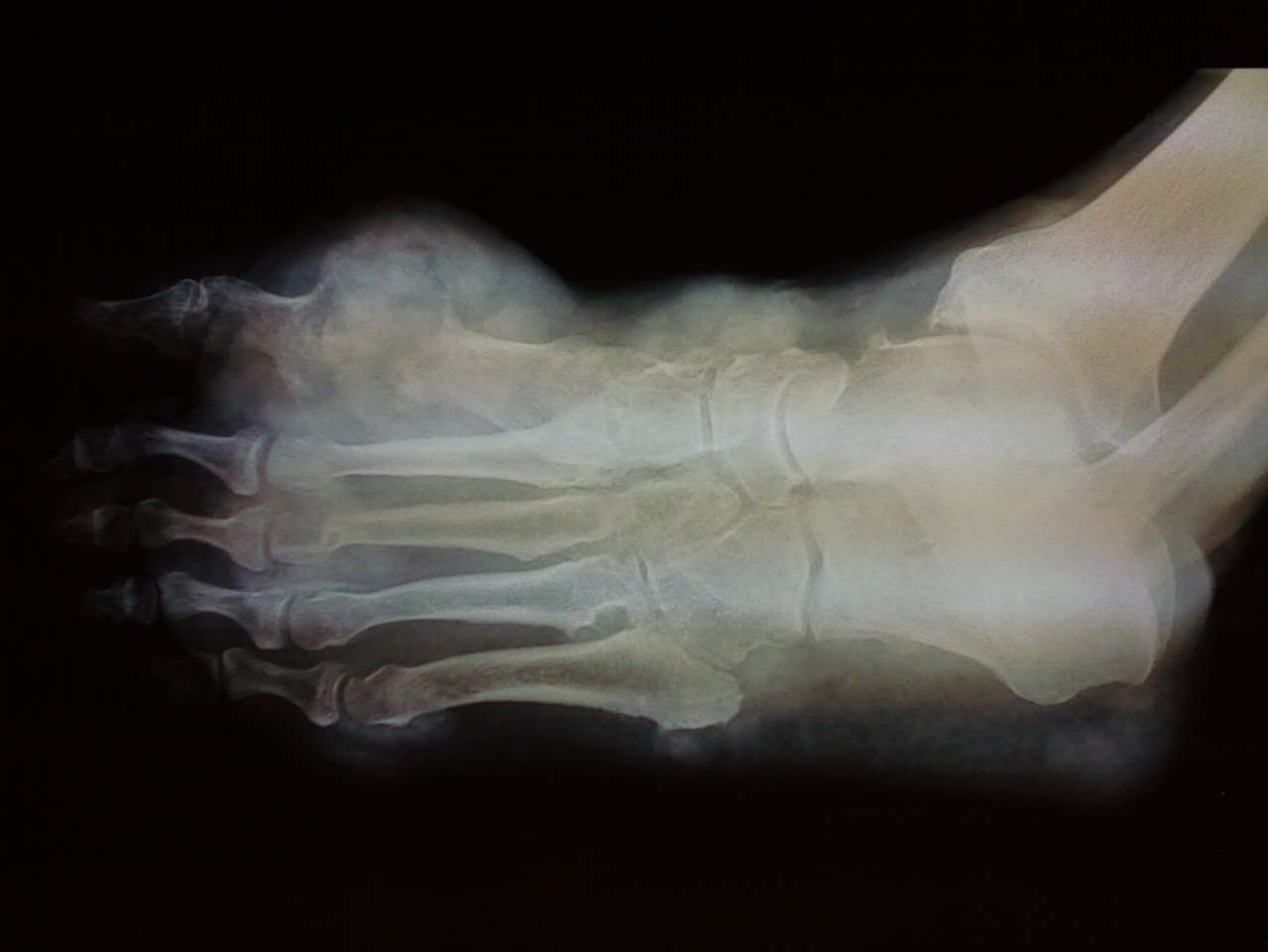
**Radiographs of the foot**. Total destruction of the first metatarsophalangeal joint and soft tissue swelling is shown as is focal involvement of dorsal and plantar surface of the foot (panniculitis).

Laboratory workup revealed leukocytosis (14.524/mm^3^), elevated C-reactive protein (7.21 mg/dl) and elevated serum uric acid (14 mg/dl). Radiographs of the foot showed soft tissue swelling and total destruction of the first metatarsophalangeal joint (Figure 6). Moderate periarticular alterations were also observed in the other joints of the foot. Cultures from the ulcerated tophus were negative. Antibiotic treatment with ciprofloxacin (800 mg/day) and intravenous administration of NSAIDs (lornoxicam 16 mg/day) was initiated.

**Figure 7:**
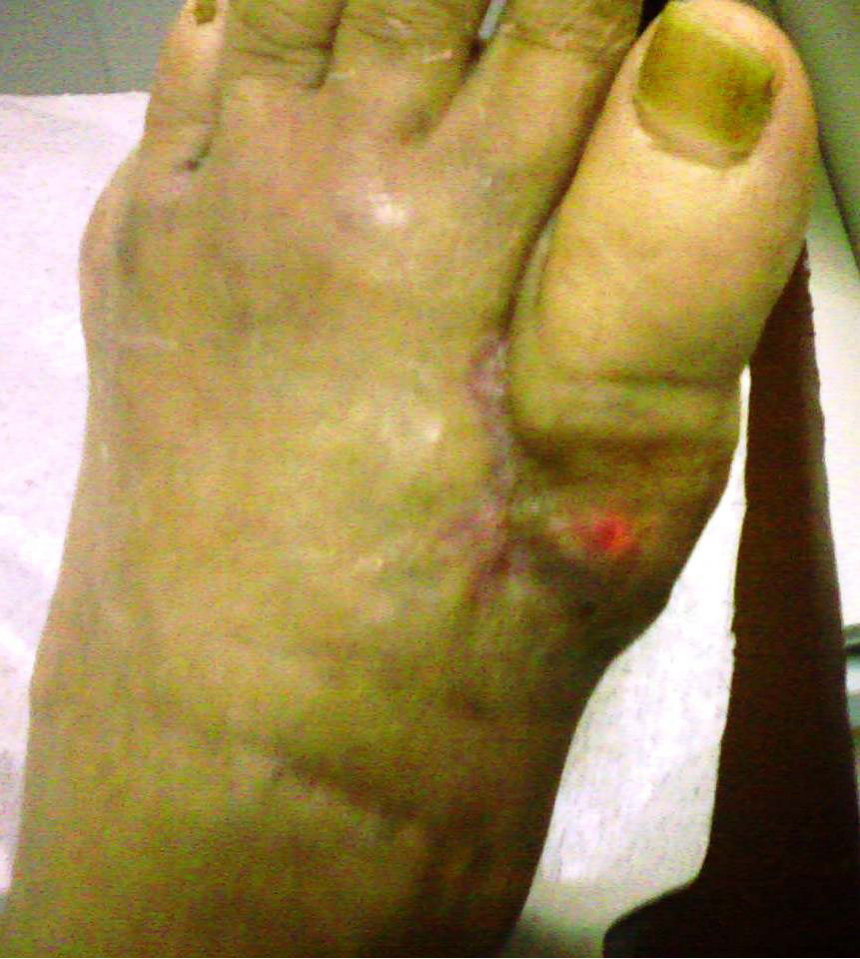
**Complete healing of the ulcer 40 days after the initial observation**.

Due to the extraordinary size of the ulcer and the complete destruction of the underlying joint, amputation of the left foot was considered. However, before resorting to this solution, a surgical debridement with lavage of the joint was performed. Debridement was also performed on the minor ulcers. Five days after admission treatment with allopurinol (300 mg/day) was initiated. The patient improved clinically and was discharged two days later. For the next 33 days foam silver-containing wound dressing (CELLOSORB^® ^Ag) and heterologous lyophilized collagen (BIOPAD^®^, equine collagen) were used on the largest of the three ulcers, on an outpatient basis, while efforts were made to keep serum uric acid levels within normal limits. All three ulcers healed completely within eight, 10 and 40 days after initial presentation, respectively (Figure 7). Six months after treatment, he remains symptom free, although he still refuses to comply with the prescribed uric acid lowering regimen and rejects any further surgical intervention.

## Discussion

Gout is the most common inflammatory arthropathy, reported to affect 2.13% of the population of the United States of America in 2009 [1]. Older age, male sex, postmenopausal state and black race are related to a higher risk for development of the disease [2]. Elevation of uric acid levels above the saturation point for urate crystal formation (6.8 mg/dl) usually results from an impaired renal uric acid excretion and although necessary, it is not sufficient to cause gout. Hyperuricemia and gout can be attributed to uric acid elevating drugs, genetic polymorphisms in genes controlling renal urate transport and predisposing dietary factors, such as consumption of red meat, seafood, alcohol and fructose containing soft beverages [3]. Other conditions associated with the disease include insulin resistance, obesity, hypertension, renal insufficiency, congestive heart failure, and organ transplantation [2].

Over time, poorly controlled gout may progress to a chronic phase, characterized by polyarticular attacks, painful symptoms between acute flares and monosodium urate crystal deposition (tophi) in soft tissues or joints [2]. Tophi are typically found on the helix of the ears, on fingers, toes, wrists and knees, on the olecranon bursae, on the Achilles tendons and also rarely on the sclerae, subconjuctivally, [4] and on the cardiac valves [5]. They can cause pain and dysfunction and are rarely associated with ulcerations [6], bone fractures [7], tendon and ligament rupture [8], carpal tunnel [9] and other nerve compression syndromes [10]. Differential diagnosis for subcutaneous or articular nodules includes septic arthritis, synovial cysts, nodal osteoarthritis, rheumatoid arthritis, sarcoidosis, lymphoma or neoplasms [11]. Synovial fluid or tophus aspiration permits diagnosis through demonstration of negatively birefringent monosodium urate crystals [2].

Treatment options for acute gouty attacks include dietary and lifestyle modifications, NSAIDs, colchicine, oral or topical steroids and corticotropin (ACTH). Interleukin-1 (IL-1) antagonists, such as anakinra, a human recombinant IL-1 receptor antagonist and canakinumab, a monoclonal antibody against IL-1β, have also shown promising results in the treatment of refractory cases or cases intolerant to classical therapy [2]. Even without treatment acute attacks usually resolve spontaneously within seven to 10 days. Normalizing hyperuricemia is of cardinal significance for the control of recurrent attacks and for the regression of tophi. This is achieved with drugs, which either favor uric acid excretion (probenecid), convert uric acid into soluble allantoin (pegloticase), or inhibit uric acid production (allopurinol, febuxostat) [2].

Surgical treatment is seldom required for gout and is usually reserved for cases of recurrent attacks with deformities, severe pain and joint destruction [11]. The main indication for surgery in patients with tophaceous gout is sepsis or infection of ulcerated tophi, followed by mechanical problems, confirmation of diagnosis and pain control [12]. Removal of tophaceous deposits from the hands can be achieved through tenosynovectomy for heavily infiltrated tendons, through a soft-tissue shaving technique for heavy skin infiltration with ulceration and draining fissures [13], or through more complex surgical approaches involving large skin incisions and excision of the tophi [14]. A hydrosurgery system applying a highly pressurized saline stream has also been used with good results for the debridement of tophi [15]. In the early stages, surgical arthroplasty can be carried out, but simple enucleation of the tophi may lead to complications such as skin necrosis, tendon and joint exposures [11]. Amputation is always a valid option for untreatable and infected ulcerations [16].

Our patient presented to the emergency department with a relatively unusual finding of ulcerated gouty tophi. Aggressive medical treatment improved hyperuricemia and facilitated the surgical approach that was initially aimed to control inflammation and avoid amputation. Heterologous, native type I collagen has a known role in tissue repair, promoting fibroblast deposition in the dermal matrix and stimulating angiogenesis, granulation tissue formation, and reepithelization. It is a valid therapeutic option in chronic wound management [17] and along with the usage of silver-containing foam dressings it resulted in an acceptable healing of the ulcer. Although the first metatarsophalangeal joint in our patient remained nonfunctional following treatment, it was able to sustain mechanical support of the foot, underlying the fact that surgical intervention should be considered in selected cases of chronic tophaceous gout.

## Consent

Written informed consent was obtained from the patient for publication of this case report and accompanying images. A copy of the written is available for review by the Editor-in-Chief of this journal.

## Competing interests

The authors declare that they have no competing interests.

## Authors' contributions

EF, ER and SM participated in the sequence alignment, researched sources for the references and drafted the manuscript. EP took the photographs and drafted the manuscript. KVB and CV helped in the interpretation of the photos and helped draft the final version of the manuscript. All authors read and approved the final manuscript.
